# Supplementary material for: Creatures of the state? Metropolitan counties compensated for state inaction in initial U.S. response to COVID-19 pandemic
Source: PLoS One. 2021 Feb 19;16(2):e0246249. doi: 10.1371/journal.pone.0246249 (PMC7894903; doi:10.1371/journal.pone.0246249)
Supplement: S1 File — (DOCX) [file pone.0246249.s001.docx]

**S1 File.**

In order to ensure that we have included all county-level orders, we conducted additional web searches based on three criteria: we checked (a) a subset of 50 randomly selected counties, (b) another subset of the 300 largest counties in the United States. We were able to confirm all 139 shelter-in-place orders that we initially included in our survey. We were then able to include an additional 14 orders among the list of largest counties (e.g., Miami-Dade County). The most common reason for these omissions was that states issued orders within a couple of days after the county’s order, which is why they were overwritten by state-level orders in the New York Times’s investigation (which only registered the most recent applicable orders for each state, including individual county orders).

Because the randomly sampled subset of counties yielded no new orders and initial results showed an overwhelming association between county size and the presence of orders, our web searches were focused on larger counties. Larger counties and cities are also more likely to be covered by national reporting. We note that our findings are entirely robust to dropping all counties that were not included in the initial database reported by the New York Times. Still, this procedure aimed at creating a complete list of shelter-in-place orders could potentially introduce sampling bias, because orders among smaller counties had a lower likelihood of being included in the dataset.

We thus conducted additional searches of counties on two lists of orders issued by cities and states. The first list is based on searches of local media reporting on non-pharmaceutical policies passed by cities and states; the second list is a curated list of local policies related to COVID-19.^^[[1]](#footnote-1)^^ We discarded interventions that were not shelter-in-place orders, such as non-binding advisories, school closings, and emergency declarations. We then confirmed all remaining orders for counties that were not already included in our dataset. The searches yielded another 8 orders from media mentions about counties (e.g., Mendocino County and several counties in Texas), and 2 orders from media mentions about large cities (e.g., Greenville, NC in Pitt county).

**S1 Table. County-level shelter-in-place orders.**

| **FIPS** | **County** | **State** | **County order** | **State order** | **Source** |
| --- | --- | --- | --- | --- | --- |
| 1073 | Jefferson County | Alabama | 23-Mar | 4-Apr | [Source](https://www.birminghamal.gov/wp-content/uploads/2020/03/2020.3.24.City-of-Birmingham.Shelter-In-Place-Ordinance.pdf) |
| 2020 | Anchorage | Alaska | 19-Mar | 28-Mar | [Source](https://www.muni.org/departments/mayor/pressreleases/documents/eo-03.pdf?fbclid=IwAR1Mq9Mpg2Xku73Zs7qTfY91ZCFKNQmn0LQPeU76RUwC_hLTwhMbFiJ90MM) |
| 6001 | Alameda County | California | 15-Mar | 19-Mar | [Source](https://www.acgov.org/documents/Final-Order-to-Shelter-In-Place.pdf) |
| 6013 | Contra Costa County | California | 15-Mar | 19-Mar | [Source](https://cchealth.org/coronavirus/pdf/HO-COVID19-SIP-0316-2020.pdf) |
| 6041 | Marin County | California | 15-Mar | 19-Mar | [Source](https://coronavirus.marinhhs.org/sites/default/files/Files/Shelter%20in%20Place/Shelter%20in%20Place%20Order%2016%20March%202020.pdf) |
| 6045 | Mendocino County | California | 17-Mar | 19-Mar | [Source](https://www.northbaybusinessjournal.com/northbay/napacounty/10831780-181/napa-sonoma-county-coronavirus-orders) |
| 6053 | Monterey County | California | 16-Mar | 19-Mar | [Source](https://www.co.monterey.ca.us/home/showdocument?id=87957) |
| 6055 | Napa County | California | 17-Mar | 19-Mar | [Source](https://www.countyofnapa.org/DocumentCenter/View/16687/3-18-2020-Shelter-at-Home-Order) |
| 6075 | San Francisco County | California | 15-Mar | 19-Mar | [Source](https://www.sfdph.org/dph/alerts/files/C19-07f-Shelter-in-Place-Health-Order.pdf) |
| 6079 | San Luis Obispo County | California | 17-Mar | 19-Mar | [Source](https://www.emergencyslo.org/en/resources/Current-Emergency-Information/Documents/Local-Emergency-Order-and-Regulation-No.-4-COVID-19.pdf) |
| 6081 | San Mateo County | California | 15-Mar | 19-Mar | [Source](https://www.smcgov.org/sites/smcgov.org/files/HO%20Order%20Shelter%20in%20Place%2020200316.pdf) |
| 6085 | Santa Clara County | California | 15-Mar | 19-Mar | [Source](https://www.sccgov.org/sites/covid19/Pages/order-health-officer-031620.aspx#:~:text=This%20Order%20shall%20become%20effective,writing%20by%20the%20Health%20Officer.) |
| 6087 | Santa Cruz County | California | 15-Mar | 19-Mar | [Source](https://www.santacruzhealth.org/Portals/7/Pdfs/Coronavirus/Shelter%20in%20Place%20Order%20March%2016%202020.pdf) |
| 6095 | Solano County | California | 17-Mar | 19-Mar | [Source](https://www.solanocounty.com/civicax/filebank/blobdload.aspx?BlobID=31828) |
| 6097 | Sonoma County | California | 16-Mar | 19-Mar | [Source](https://socoemergency.org/order-of-the-health-officer-shelter-in-place/) |
| 6113 | Yolo County | California | 17-Mar | 19-Mar | [Source](https://www.yolocounty.org/home/showdocument?id=62368) |
| 8001 | Adams County | Colorado | 24-Mar | 26-Mar | [Source](https://www.arapahoegov.com/DocumentCenter/View/9607/Press-Release-Stay-At-Home-Order-FINAL-3242020?bidId=) |
| 8007 | Archuleta County | Colorado | 22-Mar | 26-Mar | [Source](https://durangoherald.com/articles/319129) |
| 8013 | Boulder County | Colorado | 24-Mar | 26-Mar | [Source](https://assets.bouldercounty.org/wp-content/uploads/2020/05/BoulderCounty-Stay-at-Home-Order-032520.pdf) |
| 8031 | Denver County | Colorado | 22-Mar | 26-Mar | [Source](https://www.denverpost.com/2020/03/23/full-stay-at-home-order-denver-hancock/) |
| 8067 | La Plata County | Colorado | 22-Mar | 26-Mar | [Source](https://durangoherald.com/articles/319129) |
| 8097 | Pitkin County | Colorado | 22-Mar | 26-Mar | [Source](https://kdvr.com/wp-content/uploads/sites/11/2020/03/PC-3rd-Amended-Public-Health-Standing-Order_Draft-v2-1-signed.pdf) |
| 12001 | Alachua County | Florida | 22-Mar | 3-Apr | [Source](https://alachuacounty.us/Depts/Communications/Documents/ADACompliant/Stayathomeorder20-09.pdf) |
| 12011 | Broward County | Florida | 25-Mar | 3-Apr | [Source](https://www.broward.org/CoronaVirus/Documents/BC-EmergencyOrder20-03.pdf) |
| 12035 | Flagler County | Florida | 21-Mar | 3-Apr | [Source](http://www.flaglercounty.org/document_center/2020%20News%20Releases/March/EO%202020-01%20Emergency%20Beach%20and%20Park%20Facilities%20Closure.pdf) |
| 12057 | Hillsborough County | Florida | 25-Mar | 3-Apr | [source](https://www.hillsboroughcounty.org/en/media-center/press-releases/2020/03/27/hillsborough-county-emergency-policy-group-approves-safer-at-home-order) |
| 12073 | Leon County | Florida | 24-Mar | 3-Apr | [Source](https://gray-arc-content.s3.amazonaws.com/WCTV/Amended_Proclamation_Declaring_a_Continuing_State_of_Emergency.pdf) |
| 12086 | Miami-Dade County | Florida | 25-Mar | 3-Apr | [Source](https://www.miamidade.gov/information/library/coronavirus-emergency-order-safer-at-home.pdf) |
| 12087 | Monroe County | Florida | 29-Mar | 3-Apr | [Source](https://www.flgov.com/wp-content/uploads/orders/2020/EO_20-89.pdf) |
| 12095 | Orange County | Florida | 23-Mar | 3-Apr | [Source](https://www.ocfl.net/Portals/0/Library/Emergency-Safety/docs/coronavirus/Emergency%20Executive%20Order%20No.%202020-04%20-%2003-24-20.pdf) |
| 12097 | Osceola County | Florida | 25-Mar | 3-Apr | [Source](https://www.osceola.org/core/fileparse.php/7578/urlt/032520_Executive-Order-2-Shelter-in-Place-Osceola.pdf) |
| 12099 | Palm Beach County | Florida | 28-Mar | 3-Apr | [Source](https://discover.pbcgov.org/PDF/COVID19/PBC-Directing-Stay-Home-Stay-Safe-Safer-at-Home-Policy-Amendment.pdf) |
| 12103 | Pinellas County | Florida | 24-Mar | 3-Apr | [Source](http://www.pinellascounty.org/emergency/PDF/covid19/res20-20.pdf) |
| 13045 | Carroll County | Georgia | 23-Mar | 3-Apr | [Source](https://www.carrollcountyga.com/DocumentCenter/View/3515/FINAL-Carroll-County-Board-of-Health---Shelter-in-Place-3-24-20?fbclid=IwAR3LG93nhPgRz4VDSeCdxGFEczs9fZ6sgmg_UWQjbMrRhWc3zPyCfb-s3OQ) |
| 13051 | Chatham County | Georgia | 24-Mar | 3-Apr | [Source](https://www.savannahga.gov/DocumentCenter/View/18659/Shelter-At-home-Order?bidId=) |
| 13059 | Clarke County | Georgia | 19-Mar | 3-Apr | [Source](https://www.onlineathens.com/news/20200320/sheltering-in-place-social-distancing-now-law-in-athens) |
| 13063 | Clayton County | Georgia | 30-Mar | 3-Apr | [Source](https://www.news-daily.com/news/unincorporated-clayton-county-under-covid-19-shelter-in-place-order-as-of-12-01-a/pdf_e4b73d78-73a8-11ea-8500-ff415f3a95d3.html) |
| 13067 | Cobb County | Georgia | 23-Mar | 3-Apr | [Source](https://s3.us-west-2.amazonaws.com/cobbcounty.org.if-us-west-2/prod/2020-03/Declaration%20of%20Emergency.pdf) |
| 13089 | DeKalb County | Georgia | 26-Mar | 3-Apr | [Source](https://www.dekalbcountyga.gov/sites/default/files/users/user715/Stay%20at%20Home%20Order%20FINAL.pdf) |
| 13095 | Dougherty County | Georgia | 20-Mar | 3-Apr | [Source](https://wfxl.com/news/local/dougherty-county-leaders-issued-shelter-in-place-order-amidst-covid-19-pandemic) |
| 13097 | Douglas County | Georgia | 24-Mar | 3-Apr | [Source](https://patch.com/georgia/douglasville/county-health-board-debates-coronavirus-restrictions-icu-fills) |
| 13099 | Early County | Georgia | 23-Mar | 3-Apr | [Source](https://www.dothanfirst.com/wp-content/uploads/sites/33/2020/03/Signed-Declaration-3-24-20-1.pdf) |
| 13111 | Fannin County | Georgia | 22-Mar | 3-Apr | [Source](https://www.fannincountyga.com/wp-content/uploads/2020/03/Emergency-Declaration-2020.pdf) |
| 13121 | Fulton County | Georgia | 30-Mar | 3-Apr | [Source](https://fultoncountyboh.org/boh/media/attachments/2020/04/01/20.03.31.-fulton-county-boh-administrative-order.pdf) |
| 13123 | Gilmer County | Georgia | 22-Mar | 3-Apr | [Source](https://www.timescourier.com/local/gilmer-ordered-shelter-place) |
| 13135 | Gwinnett County | Georgia | 26-Mar | 3-Apr | [Source](https://www.gwinnettcounty.com/static/departments/boc/pdf/Local%20Emergency%20Order%201-4%203%2027%2020.pdf) |
| 13139 | Hall County | Georgia | 31-Mar | 3-Apr | [Source](https://7b1de02a7bcf9c1df487-6849f9022b05f72b83236695aa4e9a0a.ssl.cf2.rackcdn.com/uploads/files/2020/04/signed-resolution-stay-at-home.pdf) |
| 13227 | Pickens County | Georgia | 23-Mar | 3-Apr | [Source](https://www.pickensprogress.com/2015/news/recent-stories-index/5889-pickens-local-state-of-emergency-declaration) |
| 13241 | Rabun County | Georgia | 22-Mar | 3-Apr | [Source](https://rabun.fetchyournews.com/2020/03/23/kemp-public-health-emergency-quarantine-gema/) |
| 16013 | Blaine County | Idaho | 19-Mar | 25-Mar | [Source](https://www.co.blaine.id.us/DocumentCenter/View/11086/20-March-2020-Self-Isolation-Order-Press-Release-Blaine-County?bidId=) |
| 20091 | Johnson County | Kansas | 21-Mar | 30-Mar | [Source](https://www.jocogov.org/sites/default/files/documents/CMO/JoCo%20Public%20Health%20Officer%20Stay%20at%20Home%20Order%203-22-20.pdf) |
| 20173 | Sedgwick County | Kansas | 22-Mar | 30-Mar | [Source](https://www.sedgwickcounty.org/media/56915/covid-19-order-for-stay-at-home.pdf) |
| 23005 | Cumberland County | Maine | 24-Mar | 2-Apr | [Source](https://www.maine.gov/governor/mills/sites/maine.gov.governor.mills/files/inline-files/CORRECTED_An%20Order%20Regarding%20Further%20Restrictions%20on%20Public%20Contact%20and%20Movement%2C%20Schools%2C%20Vehicle%20Travel%20and%20Retail%20Business%20Operations.pdf) |
| 28071 | Lafayette County | Mississippi | 21-Mar | 31-Mar | [Source](https://lafco.confit.dev/wp-content/uploads/2020/04/Lafayette-County-Resoultion-Shelter-Order.pdf) |
| 28075 | Lauderdale County | Mississippi | 30-Mar | 31-Mar | [Source](https://www.sos.ms.gov/Education-Publications/ExecutiveOrders/1465.pdf) |
| 28081 | Lee County | Mississippi | 19-Mar | 31-Mar | [Source](http://www.lee.ga.us/publicnotice/files/COVID_Executive_Order_032020.pdf) |
| 29019 | Boone County | Missouri | 23-Mar | 6-Apr | [Source](https://www.showmeboone.com/common/pdf/Boone%20County%20Order%20No%202020-03C.pdf) |
| 29021 | Buchanan County | Missouri | 22-Mar | 6-Apr | [Source](https://www.stjoemo.info/DocumentCenter/View/11348/Reissued-Second-Amended-Declaration-and-Order-PDF) |
| 29037 | Cass County | Missouri | 22-Mar | 6-Apr | [Source](https://www.casscounty.com/DocumentCenter/View/2253/20200323-Cass-County-Public-Health-Emergency-Order?bidId=) |
| 29047 | Clay County | Missouri | 21-Mar | 6-Apr | [Source](https://htv-prod-media.s3.amazonaws.com/files/clay-county-public-health-emergency-order-updated-3-22-20-1584907629.pdf) |
| 29051 | Cole County | Missouri | 26-Mar | 6-Apr | [Source](https://www.colecounty.org/DocumentCenter/View/5181/Cole-County-Health-Department-Stay-at-Home-Order-3-27-2020-PDF) |
| 29077 | Greene County | Missouri | 23-Mar | 6-Apr | [Source](https://greenecountymo.gov/files/PDF/file.pdf?id=35369) |
| 29095 | Jackson County | Missouri | 23-Mar | 6-Apr | [Source](https://www.jacksongov.org/DocumentCenter/View/6660/Jackson-County-Stay-at-Home-Order) |
| 29099 | Jefferson County | Missouri | 22-Mar | 6-Apr | [Source](https://www.fordharrison.com/webfiles/Jefferson%20County%20joint%20order%20and%20press%20release.pdf) |
| 29165 | Platte County | Missouri | 21-Mar | 6-Apr | [Source](https://parkvillemo.gov/download/PlatteCountyOrder_COVID19_StayatHome.pdf) |
| 29175 | Randolph County | Missouri | 24-Mar | 6-Apr | [Source](https://www.moberlymonitor.com/news/20200324/randolph-county-issues-stay-at-home-order) |
| 29177 | Ray County | Missouri | 23-Mar | 6-Apr | [Source](https://raycountymo.com/wp-content/uploads/2020/03/RayCounty-CourtOrder-032420.pdf) |
| 29183 | St. Charles County | Missouri | 22-Mar | 6-Apr | [Source](https://www.sccmo.org/DocumentCenter/View/15365/20-06-Executive-Order-PDF) |
| 29189 | St. Louis County | Missouri | 20-Mar | 6-Apr | [Source](https://stlcorona.com/news/press-release-3212020/) |
| 29510 | St. Louis city | Missouri | 20-Mar | 6-Apr | [Source](https://assets.documentcloud.org/documents/6816639/St-Louis-City-Order-Health-Commissioner-s-Order.pdf) |
| 37021 | Buncombe County | North Carolina | 24-Mar | 30-Mar | [Source](https://files.nc.gov/doi/documents/files/COVID19/buncombe-county-of-stay-at-home-order.pdf) |
| 37025 | Cabarrus County | North Carolina | 25-Mar | 30-Mar | [Source](https://cabarruscountync.sharepoint.com/CabarrusCounty/Docs/Public-Hearing-Notice/326-Emergency-Stay-Home-Proclamation-2020.pdf?originalPath=aHR0cHM6Ly9jYWJhcnJ1c2NvdW50eW5jLnNoYXJlcG9pbnQuY29tLzpiOi9nL0NhYmFycnVzQ291bnR5L0VUQUhMT1R1ZDZWS3R4SUpiQTlwUzBnQi1TY0RSLWxYMElMeGFGVzBYeXh2clE_cnRpbWU9eWNibGtaTl8yRWc) |
| 37119 | Mecklenburg County | North Carolina | 25-Mar | 30-Mar | [Source](https://www.mecknc.gov/news/Documents/Mecklenburg%20County%20Stay%20at%20Home%20Orders.pdf) |
| 37147 | Pitt County | North Carolina | 29-Mar | 30-Mar | [Source](https://www.greenvilleonline.com/story/news/local/greenville/downtown/2020/03/30/coronavirus-greenville-stay-home-require-6-feet-distance-close-nonessential-business/5089580002/) |
| 37183 | Wake County | North Carolina | 25-Mar | 30-Mar | [Source](http://www.wakegov.com/c19docs/Documents/FinalSignedSOE3-26-20.pdf) |
| 40017 | Canadian County | Oklahoma | 28-Mar |  | [Source](https://www.okc.gov/Home/Components/News/News/3321/18) |
| 40027 | Cleveland County | Oklahoma | 25-Mar |  | [Source](http://www.oudaily.com/news/norman-mayor-breea-clark-to-announce-shelter-in-place-order-for-city-of-norman-amid/article_fb01b4f0-6df4-11ea-8a7e-23d96e5719c7.html) |
| 40109 | Oklahoma County | Oklahoma | 28-Mar |  | [Source](https://www.okc.gov/Home/Components/News/News/3321/18) |
| 40113 | Osage County | Oklahoma | 28-Mar |  | [Source](https://www.okc.gov/Home/Components/News/News/3321/18) |
| 40119 | Payne County | Oklahoma | 29-Mar |  | [Source](https://www.stwnewspress.com/covid-19/shelter-in-place-for-stillwater-going-into-effect-just-before-midnight/article_245f9bd0-72b6-11ea-8fc8-63cef71d9e5e.html) |
| 40125 | Pottawatomie County | Oklahoma | 2-Apr |  | [Source](https://www.news-star.com/story/news/2020/04/03/pott-county-commissioners-enact-countywide-shelter-in-place-order/111699248/) |
| 40131 | Rogers County | Oklahoma | 28-Mar |  | [Source](https://www.okc.gov/Home/Components/News/News/3321/18) |
| 40143 | Tulsa County | Oklahoma | 28-Mar |  | [Source](https://www.usnews.com/news/best-states/oklahoma/articles/2020-03-28/officials-15-people-with-covid-19-in-oklahoma-have-died) |
| 40145 | Wagoner County | Oklahoma | 28-Mar |  | [Source](https://www.okc.gov/Home/Components/News/News/3321/18) |
| 42003 | Allegheny County | Pennsylvania | 22-Mar | 1-Apr | [Source](https://www.governor.pa.gov/wp-content/uploads/2020/03/03.23.20-TWW-COVID-19-Stay-at-Home-Order.pdf) |
| 42007 | Beaver County | Pennsylvania | 27-Mar | 1-Apr | [Source](https://www.governor.pa.gov/wp-content/uploads/2020/03/20200328-GOV-Stay-at-Home-Order-Amendment.pdf) |
| 42011 | Berks County | Pennsylvania | 26-Mar | 1-Apr | [Source](https://www.lackawannacounty.org/wp-content/uploads/2020/03/Gov-Wolf-Shelter-in-Place-Order.pdf) |
| 42017 | Bucks County | Pennsylvania | 22-Mar | 1-Apr | [Source](https://www.governor.pa.gov/wp-content/uploads/2020/03/03.23.20-TWW-COVID-19-Stay-at-Home-Order.pdf) |
| 42019 | Butler County | Pennsylvania | 26-Mar | 1-Apr | [Source](https://www.lackawannacounty.org/wp-content/uploads/2020/03/Gov-Wolf-Shelter-in-Place-Order.pdf) |
| 42025 | Carbon County | Pennsylvania | 29-Mar | 1-Apr | [Source](https://www.governor.pa.gov/wp-content/uploads/2020/03/20200330-SOH-Stay-at-Home-Order-Amendment.pdf) |
| 42027 | Centre County | Pennsylvania | 27-Mar | 1-Apr | [Source](https://www.governor.pa.gov/wp-content/uploads/2020/03/20200328-GOV-Stay-at-Home-Order-Amendment.pdf) |
| 42029 | Chester County | Pennsylvania | 22-Mar | 1-Apr | [Source](https://www.governor.pa.gov/wp-content/uploads/2020/03/03.23.20-TWW-COVID-19-Stay-at-Home-Order.pdf) |
| 42041 | Cumberland County | Pennsylvania | 29-Mar | 1-Apr | [Source](https://www.ccpa.net/4796/Stay-At-Home-Order) |
| 42043 | Dauphin County | Pennsylvania | 29-Mar | 1-Apr | [Source](https://www.dauphincounty.org/news_detail_T14_R288.php) |
| 42045 | Delaware County | Pennsylvania | 22-Mar | 1-Apr | [Source](https://www.governor.pa.gov/wp-content/uploads/2020/03/03.23.20-TWW-COVID-19-Stay-at-Home-Order.pdf) |
| 42049 | Erie County | Pennsylvania | 23-Mar | 1-Apr | [Source](https://www.governor.pa.gov/newsroom/governor-wolf-and-health-secretary-expand-stay-at-home-order-to-erie-county-to-mitigate-spread-of-covid-19/) |
| 42069 | Lackawanna County | Pennsylvania | 26-Mar | 1-Apr | [Source](https://www.lackawannacounty.org/wp-content/uploads/2020/03/Gov-Wolf-Shelter-in-Place-Order.pdf) |
| 42071 | Lancaster County | Pennsylvania | 26-Mar | 1-Apr | [Source](https://www.lackawannacounty.org/wp-content/uploads/2020/03/Gov-Wolf-Shelter-in-Place-Order.pdf) |
| 42077 | Lehigh County | Pennsylvania | 24-Mar | 1-Apr | [Source](https://www.lehighvalleylive.com/coronavirus/2020/03/pennsylvania-stay-at-home-order-expected-in-lehigh-valley-as-coronavirus-spreads.html) |
| 42079 | Luzerne County | Pennsylvania | 26-Mar | 1-Apr | [Source](https://www.lackawannacounty.org/wp-content/uploads/2020/03/Gov-Wolf-Shelter-in-Place-Order.pdf) |
| 42089 | Monroe County | Pennsylvania | 22-Mar | 1-Apr | [Source](https://www.governor.pa.gov/wp-content/uploads/2020/03/03.23.20-TWW-COVID-19-Stay-at-Home-Order.pdf) |
| 42091 | Montgomery County | Pennsylvania | 22-Mar | 1-Apr | [Source](https://www.governor.pa.gov/wp-content/uploads/2020/03/03.23.20-TWW-COVID-19-Stay-at-Home-Order.pdf) |
| 42095 | Northampton County | Pennsylvania | 24-Mar | 1-Apr | [Source](https://www.lehighvalleylive.com/coronavirus/2020/03/pennsylvania-stay-at-home-order-expected-in-lehigh-valley-as-coronavirus-spreads.html) |
| 42101 | Philadelphia County | Pennsylvania | 22-Mar | 1-Apr | [Source](https://www.phila.gov/media/20200322130746/Order-2-Business-And-Congregation-Prohibition-Stay-At-Home.pdf) |
| 42103 | Pike County | Pennsylvania | 26-Mar | 1-Apr | [Source](https://www.lackawannacounty.org/wp-content/uploads/2020/03/Gov-Wolf-Shelter-in-Place-Order.pdf) |
| 42107 | Schuylkill County | Pennsylvania | 29-Mar | 1-Apr | [Source](https://www.governor.pa.gov/newsroom/gov-wolf-and-sec-of-health-expand-stay-at-home-order-to-carbon-cumberland-dauphin-and-schuylkill-counties-extend-school-closures-indefinitely/#:~:text=Rachel%20Levine%20revised%20their%20%E2%80%9CStay,will%20continue%20until%20April%2030.) |
| 42125 | Washington County | Pennsylvania | 27-Mar | 1-Apr | [Source](https://www.governor.pa.gov/wp-content/uploads/2020/03/20200328-GOV-Stay-at-Home-Order-Amendment.pdf) |
| 42127 | Wayne County | Pennsylvania | 26-Mar | 1-Apr | [Source](https://www.lackawannacounty.org/wp-content/uploads/2020/03/Gov-Wolf-Shelter-in-Place-Order.pdf) |
| 42129 | Westmoreland County | Pennsylvania | 26-Mar | 1-Apr | [Source](https://www.lackawannacounty.org/wp-content/uploads/2020/03/Gov-Wolf-Shelter-in-Place-Order.pdf) |
| 42133 | York County | Pennsylvania | 26-Mar | 1-Apr | [Source](https://www.lackawannacounty.org/wp-content/uploads/2020/03/Gov-Wolf-Shelter-in-Place-Order.pdf) |
| 45019 | Charleston County | South Carolina | 23-Mar | 7-Apr | [Source](https://www.scribd.com/document/453080699/Charleston-Shelter-in-Place-Ordinance-March-24) |
| 45063 | Lexington County | South Carolina | 28-Mar | 7-Apr | [Source](https://governor.sc.gov/sites/default/files/Documents/Executive-Orders/2020-04-06%20eFILED%20Executive%20Order%20No.%202020-21%20-%20Stay%20at%20Home%20or%20Work%20Order.pdf) |
| 45079 | Richland County | South Carolina | 28-Mar | 7-Apr | [Source](https://governor.sc.gov/sites/default/files/Documents/Executive-Orders/2020-04-06%20eFILED%20Executive%20Order%20No.%202020-21%20-%20Stay%20at%20Home%20or%20Work%20Order.pdf) |
| 47037 | Davidson County | Tennessee | 21-Mar | 1-Apr | [Source](https://clarksvillenow.com/local/davidson-county-institutes-safer-at-home-what-does-this-mean-for-residents/) |
| 47093 | Knox County | Tennessee | 22-Mar | 1-Apr | [Source](https://knoxvilletn.gov/UserFiles/Servers/Server_109478/File/MayorsOffice/Covid19/knoxcounty-safer-at-home-order.pdf) |
| 47157 | Shelby County | Tennessee | 24-Mar | 1-Apr | [Source](https://www.shelbycountytn.gov/DocumentCenter/View/36527/SCHD-Covid-19-Health-Directive) |
| 47187 | Williamson County | Tennessee | 29-Mar | 1-Apr | [Source](https://publications.tnsosfiles.com/pub/execorders/exec-orders-lee22.pdf) |
| 48007 | Aransas County | Texas | 26-Mar | 2-Apr | [Source](https://aransaspasstx.gov/DocumentCenter/View/1410/Aransas-County---Order-to-Stay-at-Home-03-27-2020) |
| 48027 | Bell County | Texas | 22-Mar | 2-Apr | [Source](http://www.ci.harker-heights.tx.us/images/Bell_County_Directive_3_-Stay_Home_Stay_Safe.pdf) |
| 48029 | Bexar County | Texas | 22-Mar | 2-Apr | [Source](https://www.bexar.org/DocumentCenter/View/26253/Executive-Order-NW-03---Judge-Nelson-Wolff---March-23-2020?bidId=) |
| 48039 | Brazoria County | Texas | 24-Mar | 2-Apr | [Source](https://www.brazoriacountytx.gov/home/showdocument?id=11837) |
| 48041 | Brazos County | Texas | 23-Mar | 2-Apr | [Source](https://www.brazoscountytx.gov/DocumentCenter/View/3087/2020-03-24-Amended-Brazos-County-Shelter-in-Place-Ordr-Under-County-Judg?bidId=) |
| 48061 | Cameron County | Texas | 22-Mar | 2-Apr | [Source](https://www.cameroncounty.us/wp-content/uploads/2020/03/03.23.2020-Third-Supplemental-Emergency-Management-Order-with-Countywide-Shelter-in-Place.pdf) |
| 48071 | Chambers County | Texas | 23-Mar | 2-Apr | [Source](https://www.co.chambers.tx.us/upload/page/0138/Executive%20Order%20-%20Stay%20Safe%20-%203-24-20.pdf) |
| 48085 | Collin County | Texas | 23-Mar | 2-Apr | [Source](https://www.collincountytx.gov/public_information/news/Documents/20200324%20Chris%20Hill%20Executive%20Order%20FINAL.pdf) |
| 48113 | Dallas County | Texas | 21-Mar | 2-Apr | [Source](https://www.dallascounty.org/Assets/uploads/docs/judge-jenkins/covid-19/03232020-AmendedOrder.pdf) |
| 48121 | Denton County | Texas | 23-Mar | 2-Apr | [Source](https://www.documentcloud.org/documents/6818333-EO-COVID19-032420-Signed-FINAL.html) |
| 48141 | El Paso County | Texas | 23-Mar | 2-Apr | [Source](http://epstrong.org/documents/covid19/FINAL%20to%20use%20County%20Order%207%203.24.19.pdf) |
| 48139 | Ellis County | Texas | 23-Mar | 2-Apr | [Source](https://co.ellis.tx.us/DocumentCenter/View/11040/Ellis-County-Stay-Home-Stay-Safe-Order) |
| 48157 | Fort Bend County | Texas | 23-Mar | 2-Apr | [Source](https://www.fortbendcountytx.gov/home/showdocument?id=53583) |
| 48167 | Galveston County | Texas | 22-Mar | 2-Apr | [Source](http://www.galvestoncountytx.gov/CJ/Documents/Stay%20at%20Home%20Order%20-3-23-2020.pdf) |
| 48183 | Gregg County | Texas | 24-Mar | 2-Apr | [Source](https://www.co.gregg.tx.us/sites/default/files/files/GCCOVID19%20Amended%20Declaration.pdf) |
| 48201 | Harris County | Texas | 23-Mar | 2-Apr | [Source](https://agenda.harriscountytx.gov/2020/03-24-20StayHomeWorkSafe.pdf) |
| 48209 | Hays County | Texas | 24-Mar | 2-Apr | [Source](https://hayscountytx.com/download/commissioners_court/county_judge/COVID19_3rd_Order_StayatHome.WorkSafe_Hays-County_March-25-2020_2.pdf) |
| 48215 | Hidalgo County | Texas | 25-Mar | 2-Apr | [Source](https://www.hidalgocounty.us/DocumentCenter/View/36748/3252020-Final-Modified-Emergency-Orders) |
| 48231 | Hunt County | Texas | 22-Mar | 2-Apr | [Source](http://www.huntcounty.net/upload/page/9857/docs/Disaster%20Resolution%20STAY%20IN%20PLACE.pdf) |
| 48257 | Kaufman County | Texas | 23-Mar | 2-Apr | [Source](https://drive.google.com/file/d/14ck2TCbS94GRIL2mwmbxndcrSXLtzcsJ/view) |
| 48291 | Liberty County | Texas | 23-Mar | 2-Apr | [Source](https://newtools.cira.state.tx.us/upload/page/4833/docs/ExecutiveOrder.StaySafeEmergencyMeasures.3.24.2020.pdf) |
| 48303 | Lubbock County | Texas | 28-Mar | 2-Apr | [Source](https://ci.lubbock.tx.us/storage/images/N0bK8PC32mc5L5IP8lzcN8V3iqGTObtcJJx2SM4E.pdf) |
| 48309 | McLennan County | Texas | 23-Mar | 2-Apr | [Source](http://co.mclennan.tx.us/DocumentCenter/View/10097/Amended-Order-Directing-All-Individuals-to-Shelter-at-Their-Place-of-Residence-PDF---ADA-032420) |
| 48339 | Montgomery County | Texas | 26-Mar | 2-Apr | [Source](https://www.fox26houston.com/news/montgomery-county-issues-stay-home-stop-the-spread-order) |
| 48347 | Nacogdoches County | Texas | 28-Mar | 2-Apr | [Source](https://www.co.nacogdoches.tx.us/Covid-19/Files/20200329%20Amended%20Disaster%20Declaration.pdf?v20200329140434) |
| 48355 | Nueces County | Texas | 26-Mar | 2-Apr | [Source](https://www.nuecesco.com/Home/ShowDocument?id=24936) |
| 48373 | Polk County | Texas | 24-Mar | 2-Apr | [Source](https://www.co.polk.tx.us/upload/page/3552/Polk%20County%20Executive%20Order%20-%20Order%20to%20Stay%20Home.pdf) |
| 48375 | Potter County | Texas | 29-Mar | 2-Apr | [Source](https://www.co.potter.tx.us/upload/page/7380/docs/COVID%203.30.20.pdf) |
| 48381 | Randall County | Texas | 2-Apr | 2-Apr | [Source](https://randallcounty.com/DocumentCenter/View/1147/Order-Re-Essential-Services-ExecutedPDF) |
| 48395 | Robertson County | Texas | 24-Mar | 2-Apr | [Source](https://www.co.robertson.tx.us/upload/page/5864/docs/Robertson%20County%20Order%20-%20Shelter%20in%20Place.pdf) |
| 48397 | Rockwall County | Texas | 23-Mar | 2-Apr | [Source](https://www.rockwallcountytexas.com/DocumentCenter/View/5243/Signed-and-filed-3-24-20) |
| 48407 | San Jacinto County | Texas | 24-Mar | 2-Apr | [Source](http://www.co.san-jacinto.tx.us/upload/page/6905/EMERGENCY%20ORDER%20COVID-19.pdf) |
| 48415 | Scurry County | Texas | 25-Mar | 2-Apr | [Source](http://www.co.scurry.tx.us/upload/page/7080/Stay%20Home%20Order%20Signed.pdf) |
| 48423 | Smith County | Texas | 26-Mar | 2-Apr | [Source](https://www.smith-county.com/home/showdocument?id=9086) |
| 48427 | Starr County | Texas | 23-Mar | 2-Apr | [Source](http://www.cityofroma.net/uploads/1/2/5/0/125065169/starr_county_stay_at_home_order.pdf) |
| 48429 | Stephens County | Texas | 22-Mar | 2-Apr | [Source](http://www.co.stephens.tx.us/upload/page/2574/2020%20Home/2020_03_23_14_10_56.pdf) |
| 48439 | Tarrant County | Texas | 23-Mar | 2-Apr | [Source](http://www.tarrantcounty.com/content/dam/main/public-health/PH%20DOCUMENTS/Epi/Coronavirus/latestdocuments/3-24-20_Tarrant_County_Executive_Order.pdf) |
| 48453 | Travis County | Texas | 23-Mar | 2-Apr | [Source](https://www.austintexas.gov/sites/default/files/files/Order%2020200324-007%20-%20Stay%20Home%20-%20Work%20Safe.pdf) |
| 48479 | Webb County | Texas | 26-Mar | 2-Apr | [Source](http://www.webbcountytx.gov/DistrictClerk/Covid19/Shelter%20in%20Place%20Emergency%20Order%203-27-2020F.pdf) |
| 48485 | Wichita County | Texas | 26-Mar | 2-Apr | [Source](http://www.wichitafallstx.gov/DocumentCenter/View/33425/O-14-2020-Shelter-in-Place-Order----signed) |
| 48489 | Willacy County | Texas | 25-Mar | 2-Apr | [Source](http://www.co.willacy.tx.us/upload/page/6508/docs/Annoucements/Shelter%20In%20Place%20Order.pdf) |
| 48491 | Williamson County | Texas | 23-Mar | 2-Apr | [Source](https://www.wilco.org/Portals/0/Departments/Stay%20Home%20Stay%20Safe%20Order%20-%20Judge%20Bill%20Gravell%20Jr%20-%20COVID-19%20%28Adopted%20March%2024%2C%202020%29.pdf) |
| 49011 | Davis County | Utah | 31-Mar | 27-Mar | [Source](http://www.co.davis.ut.us/docs/librariesprovider5/covid-19/public-health-order-4-1-20/public-health-order-april-1-2020.pdf?sfvrsn=4b453153_2) |
| 49035 | Salt Lake County | Utah | 28-Mar | 27-Mar | [Source](https://slco.org/globalassets/1-site-files/health/programs/covid/pho/pho3.pdf) |
| 49043 | Summit County | Utah | 24-Mar | 27-Mar | [Source](https://www.summitcounty.org/DocumentCenter/View/10751/Joint-Public-Health-Order-2020-03-FINAL) |
| 56039 | Teton County | Wyoming | 27-Mar |  | [Source](https://www.tetoncountywy.gov/DocumentCenter/View/13628/Signed-Stay-at-Home-Order-Over-65High-Risk?bidId=) |

Note: State orders are right-censored in the event history analysis, which focuses on adoption of county level orders.

**S2 Table. Robustness of results without including time of first adoption (all).**

|  | (1) | (2) |
| --- | --- | --- |
|  | County order | Any order |
| Population (logged) | .642* | .017 |
|  | (.286) | (.183) |
| Population density | .388+ | .125 |
|  | (.207) | (.097) |
| County orders in state | .287*** | .043 |
|  | (.046) | (.028) |
| County share Democrats 2016 | -.216 | .595** |
|  | (.259) | (.197) |
| County vs. state share Democrats 2016 | .533** | -.437* |
|  | (.197) | (.184) |
| State preemption | .066 | .069 |
|  | (.135) | (.090) |
| State policy innovativeness | -.292 | .021 |
|  | (.272) | (.142) |
| Median income | .106 | .105 |
|  | (.185) | (.096) |
| Income inequality | .200+ | .018 |
|  | (.103) | (.051) |
| Families below poverty level | -.718*** | .133 |
|  | (.194) | (.082) |
| Percent high school educated | -.323 | -.017 |
|  | (.217) | (.065) |
| Percent race white | .236 | .140 |
|  | (.181) | (.095) |
| Median age | -.147 | .024 |
|  | (.102) | (.039) |
| Care physicians per capita | .091 | -.024 |
|  | (.069) | (.026) |
| Percent uninsured | .741*** | .085 |
|  | (.151) | (.073) |
| ***Time variant*** |  |  |
| Cum. cases of COVID-19 | -.615*** | -.414*** |
|  | (.105) | (.058) |
| COVID-19 growth rate | .091 | .067** |
|  | (.057) | (.022) |
| Cum. tests per capita in state | -2.175*** | -1.759*** |
|  | (.467) | (.425) |
| Observations (county-days) | 222,074 | 222,074 |
| *AIC* | 2689.09 | 36248.30 |
| *df* | 19 | 19 |

All variables X-standardized by z-scoring variable; standard errors in parentheses; + p<.1, * p<.05, ** p<.01, *** p<.001

**S3 Table. Full regression table of effects by primary political orientation of county.**

|  | (1) | (2) | (3) |
| --- | --- | --- | --- |
|  | All counties | Democratic counties | Republican counties |
| Population (logged) | .842** | 1.029** | .117 |
|  | (.309) | (.334) | (.258) |
| Population density | .510* | .128 | 1.539*** |
|  | (.230) | (.285) | (.244) |
| County orders in state | .267*** | .134** | .402*** |
|  | (.046) | (.047) | (.059) |
| County share Democrats 2016 | -.182 | -.383 | -.218 |
|  | (.256) | (.382) | (.302) |
| County vs. state share Democrats 2016 | .657** | 1.004*** | .429+ |
|  | (.202) | (.226) | (.256) |
| State preemption | .010 | .316* | -.356* |
|  | (.138) | (.157) | (.150) |
| State policy innovativeness | -.399 | -1.140+ | -.295 |
|  | (.324) | (.598) | (.336) |
| Median income | .117 | .801** | -.086 |
|  | (.183) | (.273) | (.255) |
| Income inequality | .182+ | .309+ | .009 |
|  | (.100) | (.167) | (.175) |
| Families below poverty level | -.845*** | -.327 | -.776* |
|  | (.180) | (.217) | (.317) |
| Percent high school educated | -.230 | .174 | -.363+ |
|  | (.223) | (.390) | (.211) |
| Percent race white | .144 | .305 | -.089 |
|  | (.171) | (.190) | (.256) |
| Median age | -.087 | -.115 | -.008 |
|  | (.107) | (.168) | (.182) |
| Care physicians per capita | .091 | .027 | .058 |
|  | (.084) | (.111) | (.173) |
| Percent uninsured | .779*** | .797*** | .721*** |
|  | (.160) | (.216) | (.206) |
| Date of first COVID-19 case | -.141 | -.231 | -.586*** |
|  | (.110) | (.148) | (.154) |
| Cum. cases of COVID-19 | -.906*** | -.979*** | -1.072*** |
|  | (.116) | (.119) | (.182) |
| COVID-19 growth rate | .057 | .081 | .011 |
|  | (.051) | (.051) | (.083) |
| Cum. tests per capita in state | -1.881*** | -1.235+ | -2.653*** |
|  | (.497) | (.645) | (.505) |
| Observations | 174048 | 32856 | 141192 |
| *AIC* | 2689.09 | 1110.55 | 1331.51 |
| df | 19 | 19 | 19 |

All variables X-standardized by z-scoring variable; standard errors in parentheses; + p<.1, * p<.05, ** p<.01, *** p<.001

1. These additional lists of nonpharmaceutical interventions are available here: [https://github.com/jataware/COVID-19-data](https://github.com/jataware/covid-19-data) and here: [https://COVID19.nlc.org/resources/COVID-19-local-action-tracker/](https://covid19.nlc.org/resources/covid-19-local-action-tracker/) (accessed on October 28, 2020) [↑](#footnote-ref-1)
